# Surveillance of Mortality among Atomic Bomb Survivors Living in the United States Using the National Death Index

**DOI:** 10.2188/jea.14.17

**Published:** 2005-03-18

**Authors:** Yasuyuki Fujita, Chikako Ito, Kiyohiko Mabuchi

**Affiliations:** 1 Department of Public Health, School of Medicine, Shimane University.; 2 Health Management Center, Hiroshima Atomic Bomb Causality Council.; 3 Radiation Epidemiology Branch, Division of Cancer Epidemiology and Genetics, National Cancer Institute.

**Keywords:** atomic bomb survivors, the United States of America, National Death Index, mortality

## Abstract

BACKGROUND: The National Death Index is a useful source to establish the death of an individual and to determine the cause of death. We identified deaths in atomic bomb survivors in the United States who were lost to follow-up through the National Death Index, and examined the completeness of mortality ascertainment in atomic bomb survivors in the US through the National Death Index.

METHODS: Since 1977, biennial medical examinations of atomic bomb survivors in the US have been conducted. The 1,073 atomic bomb survivors in the US included 764 individuals who had medical examinations at least once in sixteen years from 1977 through 1993 and 309 individuals who reported atomic bomb survivorship to medical examination project themselves. Of the 1,073 survivors living in the US, 471 people who participated in the ninth health examinations of atomic bomb survivors living in the US in 1993 were removed, and two people among the remaining 602 individuals had no information about their birth dates and Social Security numbers. An investigation of those deceased between 1979 and 1993 was conducted among 600 of the atomic bomb survivors in the US. Death certificates for atomic bomb survivors in the US were requested from the National Death Index. A comparison was made between the information on the death certificates acquired through the National Death Index and the data ascertained from the medical examination project conducted from 1979 through 1993.

RESULTS: Forty-nine death certificates were obtained using the National Death Index. By sex, the dominant cause of death in females was malignant neoplasm, accounting for 53%. In males, it was circulatory disease, accounting for 37%. The National Death Index and the medical examination project determined that 57 deaths had occurred between 1979 and 1993. The sensitivity and specificity of the National Death Index is 86% and 97% respectively.

CONCLUSION: It is suggested that the National Death Index is useful to follow up mortality among atomic bomb survivors in the US.

Health examinations of atomic bomb (A-bomb) survivors living in the United States were conducted in 1977 for the first time and have been continued to date.^1^^-^^4^ This project started as a joint project of the Hiroshima Prefectural Medical Association and the Radiation Effects Research Foundation with the support of the Ministry of Health and Welfare of the Japanese government. The project has also received financial support from Hiroshima Prefecture, Hiroshima City, and the Hiroshima Atomic Bomb Casualty Council. Examinees are, in principle, the individuals who were directly affected by the bomb in Hiroshima City or Nagasaki City or neighboring areas, those who entered the area within about 2 km from the center of the blast within two weeks after the atomic blast, or those who disposed of the victim’s bodies or rescued victims. Also included are the children of those mentioned, identified as individuals with at least one parent who possesses an A-bomb survivor’s health handbook. Examinees were given a physical examination, clinical laboratory tests, and cancer screening.

The National Death Index is useful because deaths can be confirmed for those whose deaths have not been identified. The National Death Index has in its database information on those who have died in the US since 1979.^5^ Death information retrieval by the National Death Index has been used for many epidemiologic studies.^6^^-^^8^

The follow-up study of A-bomb survivors in the US is important because it investigates the health effects of A-bomb radiation and it is carried out to assess an interaction between radiation and socioeconomic or lifestyle risk factors. Nearly all deaths of A-bomb survivors in Japan have been identified from the obligatory family registration system (Koseki). However, there is no family registration system in the US. It is not always accurate to ascertain the survival status from the medical examination services based on the report from proxy or surrogate respondents. We applied to the National Death Index for the use of its database to follow up the participants of the health examinations and succeeded in obtaining a permit for the use (NDI Appl. #94-0013).

In this study, we identified deaths in A-bomb survivors in the US who were lost to follow-up through the National Death Index and we examined the completeness of mortality ascertainment in A-bomb survivors in the US through the National Death Index.

## METHODS

Since 1977, the biennial medical examinations of A-bomb survivors in the US have been conducted. The 9th health examination program lasted thirty days from June and July 1993. The 1,073 A-bomb survivors in the US included 764 individuals who had medical examinations at least once in the 16 years from 1977 through 1993 and 309 individuals who reported A-bomb survivorship to medical examination project themselves. Of the 1,073 survivors living in the US, 471 people who participated in the 9th health examinations of A-bomb survivors living in the US were removed, and two people among the remaining 602 individuals had no information about their birth dates and Social Security numbers. Then, 600 individuals were considered for retrieval of death information from the National Death Index. The period considered for the study was the 15 years from 1979 through 1993 inclusive.

A list of the subjects for information retrieval including their names (last name, first name, middle initial), Social Security numbers, dates of birth, father’s surname, age at death, sex, race, and State of residence was sent to the National Death Index. Marital status and state of birth, which are requested by the National Death Index, were not included in the list because no information on these two was available. The items including last name, first name, middle initial, Social Security number, dates of birth, father’s surname and sex, are used in searching the National Death Index file. Little time is required for information retrieval by the National Death Index, and the NDI Retrieval Report was sent back promptly. The number of batches and the duration of retrieval work, and the number of retrievals are used to calculate the charges to the National Death Index.

The possible NDI record matches with items of state of death, death certificate number, and date of death are listed on the NDI Retrieval Report. The individuals on the NDI Retrieval Report with possible NDI record matches sent by the National Death Index, and the A-bomb survivors living in the US were matched by name, Social Security number, date of birth, father’s surname, age at death, sex, race, state of death and those with a high matching rate were selected by visual inspection as possibly dead.

Requests for the death certificates of the individuals possibly dead were submitted, together with the information required on decedents (state file number, date of death, name of decedent) and other documents required and handling fees, to the department of health statistics of the state in which the individuals of interest used to live.

Death certificates were obtained from the department of health statistics of the state in which the individuals died. Causes of death were coded according to the 9th revision of the International Classification of Diseases and Injuries (ICD), and the selection of underlying causes of death was based on the World Health Organization rule.^9^ If an A-bomb survivor in the US was deceased, family members or friends reported the survivor’s death to the medical examination services of A-bomb survivors in the US. The validity of the death information retrieval by the National Death Index was examined by comparing the dead confirmed through the services of medical examinations of A-bomb survivors living in the US with the dead searched by the National Death Index.

All documents received from the National Death Index and copies of death records were kept in locked files until necessary information had been coded and entered into computer files. Documents in the locked files were only accessible to the authorized project staff. Documents containing identifying information were shredded after all information had been processed and entered into computer files. Computer files contained no personally identifiable information. This study was approved by the Human Investigation Committee of the Collaborative Research Project on Atomic Bomb Causalities in 1994.

## RESULTS

Mortality among 600 A-bomb survivors with unknown vital status in the US for 1979 through 1993 was ascertained by searching the National Death Index. The average age of A-bomb survivors in the US was 50.5 years in 1979 (standard deviation [SD] = 10.7 years, range 33 - 91 years). The survivors included 150 men (average age 49.6 years in 1979, SD = 10.4 years, range 33 - 82 years) and 450 women (average age 50.9 years in 1979, SD = 10.8 years, range 33 - 91 years). One hundred percent of persons with items such as last name, first name, sex, birth date, and race were provided to the National Death Index. Fifty percent of persons with Social Security numbers and 19% with a middle name initial were provided to the National Death Index.

The NDI record was searched for 600 A-bomb survivors in the US. The list of 262 possible NDI records for 104 A-bomb survivors was sent from the National Death Index. An A-bomb survivor has one or more NDI records. We determined one NDI record with the highest possibility in matches based on any of the matching items among plural NDI records for one individual by visual inspection. Of 262 NDI records, 104 were considered worthy of further investigation by visual inspection. One hundred and fifty-eight NDI records were eliminated from further investigation. Of the 104 NDI records, 67 were considered worthy of further investigation using death certificates. Thirty-seven NDI records were eliminated from further consideration. Of the 67 NDI records, for which we received replies from the department of health statistics of each state in response to our applications for death certificates, 49 were matches associated with A-bomb survivors in the US. Eighteen NDI records were false matches of the A-bomb survivors in the US. Forty-nine individuals matched 6 items including first name, last name, birth year, birth month, father’s name, and sex. Eighteen individuals matched at least two items including birth year (within two years) and birth month.

For the 18 people who were not regarded as A-bomb survivors living in the US, the degree of agreement of the name, date of birth, and Social Security number and the reasons why they do not qualify are shown in Table 1. There were as many as 10 A-bomb survivors living in the US whose names did not match the names of the dead retrieved from the National Death Index. This was the most predominant reason for disqualification. The state departments of health statistics reported that they could not find the death certificates for seven A-bomb survivors. Race did not match for one person.

**Table 1.** Examination of 18 disqualified persons

No	Name			Birth Date			Social Security Number	Reason for Disqualification
	
	First	Middle	Last	Month	Day	year		
1	I	B	N	X		X	Unknown	Name
2	I	B	N	X		X	Unknown	No Death Certificate at the state department of health statistics
3	I	B	X	X		-01	Incomplete agreement	First Name, Father’s Name
4	I	B	N	X		+01	Unknown	No Death Certificate at the state department of health statistics
5	I	B	N	X		-01	Unknown	No Death Certificate at the state department of health statistics
6	I	B	N	X	X	-02	Unknown	Name
7	I	B	N	X		X	Unknown	No Death Certificate at the state department of health statistics
8	I	B	N	X		X	Unknown	Name
9	I	B	N	X		-01	Unknown	Name
10	I	B	N	X		-01	Unknown	Name
11	I	B	N	X		-01	Unknown	Name
12	I	B	N	X		-01	Unknown	Name
13	X		X	X		-01	Unknown	Caucasian
14	I	B	X	X	X	X	Incomplete Agreement	First Name, Father’s Name
15	I	B	N	X		+01	Unknown	No Death Certificate at the state department of health statistics
16	I	B	X	X		X	Unknown	No Death Certificate at the state department of health statistics
17	X		N	X		X	Disagreement	No Death Certificate at the state department of health statistics
18	I	X	N	X		-01	Incomplete Agreement	Name

Note. X : Matched exactly B : No Middle name I : Initials matched N : Matched an phonetic code -01 : Date of birth on NDI is one year early

Forty-nine deaths (average age 67.5 years at death, SD = 16.0, range 42-102) were obtained using the National Death Index. They included 19 men (average age 63.7 years at death, SD = 14.2, range 48-92) and 30 women (average age 69.8 years at death, SD = 16.9, range 42-102). The causes of death by sex are shown in Table 2. The most common cause of death for men was circulatory disease, which accounted for 37% of deaths. That for women was malignant neoplasm, which accounted for 53% of deaths.

**Table 2.** Number of deaths by cause of death

Causes of Death (ICD9)	Male	Female
Malignant Neoplasm (140-208)	6 (32)	16 (53)
Endocrinologic, Nutrition, Metabolic, and Immunological Disorders (240-279)	-	1 (3)
Neurological and Sensory Organ Diseases (320-389)	1 (5)	-
Circulatory Diseases (390-459)	7 (37)	6 (20)
Respiratory Diseases (460-519)	1 (5)	4 (13)
Digestive Diseases (520-579)	2 (11)	1 (3)
Urinary and Reproductive Diseases (580-629)	-	1 (3)
Injuries and Intoxication (E800-E999)	2 (11)	1 (3)

Total	19 (100)	30 (100)

Percentages in parentheses.

ICD: International Classification of Diseases and Injuries.

Forty-nine people, for whom death certificates were obtained by enlisting the help of the National Death Index search, were compared with the 54 people (as of January, 1995) whose deaths became known through the examinations of A-bomb survivors living in the US. Of the 57 deaths that occurred between 1979 and 1993, 33 deaths were identified both by a search of the National Death Index and the health examinations, 16 were identified by the National Death Index search only, and 8 by the health examinations only.

The sensitivity of the National Death Index in ascertaining a death among A-bomb survivors in the US between 1979 and 1993 was 86% (49/57). Sensitivity among those with Social Security number is 82% (18/22), and sensitivity among those without Social Security numbers 91% (31/34). Sensitivity among those without Social Security numbers is higher than those with Social Security number.

The specificity of the National Death Index in ascertaining that A-bomb survivors in the US were alive between 1979 and 1993 was estimated before and after visual inspection. Before visual inspection, the specificity was 90% ((600-57-18-37)/(600-57)). After visual inspection, the specificity was 97% ((600-57-18)/(600-57)). The specificity after visual inspection is significantly higher than that before visual inspection.

## DISCUSSION

Forty-nine death certificates have been obtained by the National Death Index to follow up the participants in the health examinations of A-bomb survivors living in the US and to confirm their vital status and the causes of deaths. Among them, the most common cause of death for men was circulatory disease and that for women was malignant neoplasm. The National Death Index Plus search includes the causes of death. We did not utilize the National Death Index Plus in this study, however, because a trained nosologist coded the cause of death.

The 12th health examination, in 1999, was conducted on 414 A-bomb survivors living in the US.^4^ The mean age of the examinees was 68.0 years. The number of 60-year-olds and above was 315 (76.1%). The prevalence of obesity (BMI) (men: 27 kg/m^2^ or above, women: 25 kg/m^2^ or above), hypertension [(systolic blood pressure: 160 mmHg or above, or diastolic blood pressure: 95 mmHg or above)], and hyperlipemia (triglycerides: 180 mg/dL or above) was 28.7%, 28.7%, and 21.3% respectively. These are all well-known risk factors for coronary heart disease.

Among A-bomb survivors in Hiroshima and Nagasaki, malignant neoplasm is an important health problem. According to the recent data on cancer mortality for A-bomb survivors in the Radiation Effects Research Foundation’s Life Span Study cohort, significant dose responses to radiation exposure were observed for solid cancer and leukemia.^10^^,^^11^ For solid cancer, about 25% of the excess deaths in 1950-1990 occurred during the last 5 years. For leukemia only about 3% of the excess deaths in 1950-1990 occurred in the last 5 years.^11^ Solid cancer induced by irradiation may have a longer latent period than leukemia.

Among A-bomb survivors in Hiroshima and Nagasaki, non-cancerous diseases may be another health problem. Shimizu et al.^12^ reported a significant increase in noncancerous disease death rates with radiation dose among A-bomb survivors in Japan. Increasing trends are observed for diseases of the circulatory, digestive, and respiratory systems.

As a part of the NI-HON-SAN Study,^13^ the mortality and predictive factors of coronary heart disease among men of Japanese ancestry in Japan and Hawaii were compared on the basis of 12-year follow up data using comparable methods of case ascertainment and risk factor measurement. Age-adjusted mortality rates of coronary heart disease during the 12-year follow up, were 40 percent higher (not statistically significant) among Japanese American men in Hawaii than among indigenous Japanese men.^14^ The age-adjusted mean fibrinogen level in Japanese-American men was significantly higher than in native Japanese men.^15^ Sociocultural changes may influence the cerebrovascular disease development among A-bomb survivors living in the US.

Concerning the accuracy of death information retrieval from the National Death Index, Rich-Edwards et al.^16^ reported on the validity of the National Death Index based on the 197 deaths of those aged 60-69 years in 1989 and 1,997 persons to be alive of the same age, and found that the sensitivity and the specificity were 98% and 100%, respectively. The sensitivity of 86.0% in this study is lower than that of previous studies.

Calle et al.^17^ reported that the sensitivity varied with demographic factors (race, sex) and the availability of identifying information (Social Security number, middle initial). The single most important determinant of sensitivity was Social Security number. Ninety-seven percent of known deaths were accurately identified when the Social Security number was available, versus 87 percent when it was not known. He also reported that the most common reason for no matches with the National Death Index was the use of an informal first name. Other common reasons include disagreement on birth month or year.

Of the 57 deaths that occurred between 1979 and 1993, 8 deaths were identified by the health examination only. The reasons why NDI could not identify these 8 deaths are considered as follows: (1) health examination identified these 8 deaths in error, (2) the eight deaths are not registered with the National Death Index, and (3) wrong information, including names, are registered on the database of National Death Index. It is important to re-examine these 8 deaths in names, birth dates and death dates, and to submit the 8 deaths with additional information to the National Death Index repeatedly.

The sensitivity of 91% without Social Security number is higher than that of 82% with Social Security number in this study. Social Security number in A-bomb survivors living in the US does not influence the validity of matching by the National Death Index. The specificity of 97% after visual inspection is significantly higher than that of 90% before visual inspection. Visual inspection is important for specificity. When individuals with all items provided on our record did not exactly agree with items on the NDI record, we could determine whether they were A-bomb survivors or not by exact matching the personal identification items such as the spelling of name, eight digits of Social Security number, sex, and birth date on the death certificates.

Boyle et al.^18^ reported that deaths missed by the National Death Index occurred more frequently among those with certain characteristics, such as non-white racial background, non-honorable discharge, and low rank at discharge. The National Death Index’s missed deaths differed considerably by race; 23 percent of deaths among non-whites were missed relative to only 7 percent among whites. Socioeconomic status may influence the sensitivity of the National Death Index.

Before 1979, the Social Security Administration (SSA) has been the primary source for identifying deaths in cohort studies in the US. Schnorr et al.^19^ examined the completeness of the SSA Death Master File by comparing it with the US Vital Statistics records and by searching the SSA Death Master File for known decedents from seven cohorts. Overall, only 53% of reported US deaths and 75% of known deaths in their seven cohorts were included in the SSA Death Master File. Ascertainment was better after 1975 (89-95%).

In conclusion, it is suggested that the National Death Index is useful to follow up mortality among A-bomb survivors in the US. To examine the completeness of mortality ascertainment in more detail, we need further investigation of deaths not found by the National Death Index.
